# Using the Model, Lead, and Test Technique and “GoTalk NOW” App to Teach Children With Intellectual and Developmental Delays to Correctly Request

**DOI:** 10.3389/fpsyg.2021.811510

**Published:** 2022-02-09

**Authors:** Nabil Sharaf Almalki

**Affiliations:** Department of Special Education, College of Education, King Saud University, Riyadh, Saudi Arabia

**Keywords:** GoTalk NOW app, intellectual and developmental delays, iPad, model-lead-and-test technique, Saudi Arabia

## Abstract

Augmentative and alternative communication systems have been successfully used to help pupils with intellectual and developmental disabilities for functional communication skills. Whereas the effects of the GoTalk NOW application (app) available on the iPad have been investigated, no such study has been conducted in Saudi Arabia. This study investigated the effects of the “model, lead, and test” (MLT) technique paired with the GoTalk NOW app, which is used to teach four children with intellectual and developmental delays to correctly request. The participants were four children (three boys and one girl) from 6 to 8 years of age with intellectual and developmental disabilities. A multiple baseline design was implemented across all participants. All four participants made clear progress using the GoTalk NOW app on the iPad, and implementation of the MLT technique was successful in helping the participants request correctly and independently. This study provided a practical, effective, and easy-to-use method for special education teachers to teach children with disabilities. It contributes to expand the range of teaching options for children with intellectual and developmental disabilities.

## Introduction

Communication contributes greatly to the exchange of ideas and cultures, and also social and emotional interaction with the surrounding environment. It is also an essential factor in the learning process and cognitive growth of individuals through the use of different evaluative methods (Da Fonte and Boeseh, 2016; Okoli, 2017). Limited communication is one of the most common problems experienced by individuals with intellectual disability, and this problem increases with the severity of the disability. The delay in the cognitive development of such individuals is one of the reasons for their low level of ability to communicate: they acquire language development at a slower rate, which results in delayed language development compared with their cohort (Hagan and Thompson, 2013; Paterson, 2014; Norrelgen et al., 2015). A complete loss of spoken language may result for those with severe intellectual disabilities. Even in the best of cases, the spoken language of those with intellectual disabilities is disturbed (Stephenson, 2009; Wilder et al., 2015).

Several studies have indicated that some students with developmental and intellectual disabilities suffer from a deficiency in the process of language communication, resulting in multiple problems in educating and interacting with them (Aldabas, 2017). This has necessitated the development of many augmentative and alternative communication methods (Ahlgrim-Delzell et al., 2014) to help pupils who lack spoken language skills by identifying appropriate and the most effective communication tools and approaches with the relevant educational strategies based on the needs of such individuals (Herberg et al., 2011; Ruwe et al., 2011).

Both verbal and non-verbal communication are equally important. Whereas verbal communication occurs through words, non-verbal communication involves the following: body language, speech, physical expressions, facial expressions, and eye–head–hand movements. All these components of non-verbal communication are often spontaneous and expressive of their simultaneous sincerity (Glennen and DeCoste, 1997; Glozman, 2004; Beukelman and Mirenda, 2013). Importantly, the impact of the speaker’s influence through non-verbal communication may be stronger, because the meaning received by the audience is one-third verbal and two-thirds non-verbal (Okoli, 2017).

A child who has trouble in communicating may exhibit various indicators, such as difficulty following directions, attending to dialog, pronouncing words, realizing words, or expressing themselves owing to stuttering or a hoarse voice. Language disorders might include difficulties in expressing ideas consistently, learning new vocabulary, understanding and remembering information or questions, reading, understanding or repeating spoken material, and learning the alphabet (Brice, 2001; Okoli, 2017). All of these problems or disorders can be addressed through treatment; whereas some issues can never be treated, children can nevertheless manage to learn new strategies to overcome their problems (such as those of stuttering and an attention deficit). Some children can overcome their difficulties as they get older, whereas others may compensate for direct communication by electronic means, such as through an augmentative and alternative communication device or a hearing aid (Brice, 2001).

Communication and language deficits demonstrated by people with multiple disabilities are not precisely defined and appear in several forms, including problems with speech, linguistic and verbal development, poor linguistic stock, and linguistic delay. Many people with multiple disabilities need alternative systems to communicate with the outside world (Beukelman and Mirenda, 2013; Wilder et al., 2015).

Augmentative and alternative communication includes all non-verbal communication methods for the purpose of encoding and exchanging messages. These approaches help individuals with severe communication disorders compensate for their inability to actively enhance their participation in various communicative interactions (Wilder et al., 2015; Aldabas, 2017). These methods use diverse forms of communication such as gestures, body expressions, facial expressions, symbols, images, sound output devices, and other computer-based technologies in academic and social activities at school, home, and society (Stancliffe et al., 2010; Mukhopadhyay and Nwaogu, 2013; Clinical Innovation and Governance, 2016).

High-tech systems include the use of electronic communication panels and/or speech enhancement devices. These electronic boards display communication messages using pictures, drawing lines, sentences, words, or letters. The message is activated by touch or a laser beam to produce digital phrases that are recorded using a human voice (Hanline et al., 2007; Dundon et al., 2013; Genc-Tosun and Kurt, 2017; Alzrayer et al., 2020). Devices can range from single-message audio output devices (such as a BIG Mac) to devices with multiple options placed in a network mode of display (such as an iPad with GoTalk NOW); more expensive devices such as Liberator and Vmax that require a computer with a complex software are also available. Nevertheless, it is important to conduct a comprehensive evaluation of the options to determine the most appropriate device for the person to be cared for. As technology is constantly evolving and changing, practitioners prefer to stay informed (Beukelman and Mirenda, 2013; Dundon et al., 2013; Hill and Flores, 2014; Clinical Innovation and Governance, 2016).

Augmentative and alternative communication styles play a vital role in helping pupils with intellectual and developmental disabilities acquire communication skills (Da Fonte and Boeseh, 2016). Young children with disabilities such as cerebral palsy, autism, and Down’s syndrome often have difficulty using speech to communicate (Ahlgrim-Delzell et al., 2014; Alzrayer et al., 2020). Augmentative and alternative methods, which include the use of gestures, pictures, and/or voice communication, can provide these children with the ability to communicate more effectively. Moreover, effective interventions in augmentative and alternative approaches can play an important role in supporting cognitive, communication, and social development (Thistle and McNaughton, 2015; Aldabas, 2017).

Individuals with severe intellectual disabilities often fail to use augmentative and alternative methods to acquire new vocabulary. To achieve the maximum vocabulary function, the basic vocabulary that is frequently used should be used as the core vocabulary) Stephenson, 2009(. For many users of augmentative and alternative methods, the basic vocabulary often refers to concepts than to tangible elements, and for those with severe intellectual disabilities, the primary vocabulary of augmentative and alternative methods often consists of words referred to concretely than to concepts (Snodgrass et al., 2013; Wilder et al., 2015). In recent years, the iPad has emerged as an effective device that can be widely used for children with intellectual and developmental disabilities to help them acquire many of the necessary skills (Dundon et al., 2013; Alzrayer et al., 2020). There are many software programs developed in iPad devices that help children with intellectual and developmental disabilities communicate successfully (Achmadi et al., 2012; Hill and Flores, 2014).

Teaching children with developmental and intellectual disabilities using the iPad as a tool for communication can be accomplished using direct instruction in the form of the “model, lead, and test” (MLT) error correction technique. MLT error correction involves the teacher modeling the correct response to the child, both the teacher and child responding correctly in the same task together, and then the teacher requesting the child to correctly complete the task independently. If the child completes the task correctly, then the teacher moves to another academic or behavioral task. If the child is unable to complete the task correctly, then the MLT error correction technique is applied until the child responds or completes the task independently. This technique is repeatedly implemented until the child performs the task correctly and independently, for which the child may need multiple attempts (McLaughlin et al., 2011; Dundon et al., 2013).

The use of the MLT error correction technique for children with developmental and intellectual disabilities has received some attention (Peterson et al., 2008; Bechtolt et al., 2014; Datchuk and Rodgers, 2019; Knight et al., 2019), and the iPad tablets have been proven to be effective tools in developing the communication skills of such children and also in helping them learn and perform various academic and behavioral tasks successfully (Achmadi et al., 2012; Boesch et al., 2013; Lorah et al., 2013; Hill and Flores, 2014). In two such studies (Dundon et al., 2013; Ward et al., 2013), the MLT technique was paired with the GoTalk NOW application (app) to teach children with autism and developmental delays to make requests. However, to our knowledge, no previous studies have investigated the effect of this approach on teaching children with intellectual and developmental disabilities or intellectual and developmental delays. Moreover, previous studies have tended to be conducted with single participant samples in Western settings. This study focused on pairing the use of the iPad GoTalk NOW app with the MLT technique to teach requesting skills to four Arab children with intellectual and developmental disabilities and speech impairments. This study focused on tablets or digital devices (GoTalk NOW app) on the iPad rather than on the picture exchange communication system or direct instruction flashcards (Dundon et al., 2013; Hill and Flores, 2014; Alzrayer et al., 2020). It also focused on using apps on the iPad for children with intellectual and developmental disabilities in contrast to most other studies that have focused on children with autism spectrum disorders (Ward et al., 2013; Genc-Tosun and Kurt, 2017; Vlachou and Drigas, 2017). To date, there have been no studies investigating the effects of the MLT error correction technique paired with the GoTalk NOW app (Arabic version) on an iPad to teach four children with intellectual and developmental disabilities.

The purpose of this study was to (a) evaluate the effectiveness of the MLT error correction technique paired with the GoTalk NOW app on an iPad to teach four children with intellectual and developmental disabilities to correctly request, (b) evaluate the effects of removing the MLT technique and then having the children request independently, and (c) extend the use of the MLT technique using apps on an iPad^2^ that are appropriate for augmentative and alternative communication.

## Materials and Methods

### Participants

The participants were four children (three boys and one girl) from 6 to 8 years of age with a diagnosis of intellectual and developmental disabilities. They were recruited through a special education center, located in Riyadh, the capital city of Saudi Arabia. The center provides behavioral intervention services to children with disabilities. The eligibility criteria to choose participants were (a) a diagnosis of intellectual and developmental disabilities, (b) had significant delays in communication, and (c) no prior history of using electronic devices, such as iPad tablets. The parents gave informed consent for each participant, and all the participant names are pseudonyms.

#### Ryan

Ryan was a 6-year-old boy with a diagnosis of moderate intellectual and developmental disabilities and speech impairment. The Stanford-Binet Intelligence Scales, fifth edition (Roid, 2003), was used for all participants to determine their disability. Ryan had an IQ score of 40, and his score on the adaptive Arabic version of the Preschool Language Scale-4 (PLS-4) (Abu-Hasiba, 2015) was 50 for receptive and 15 for expressive language. He could make sounds with the words *mama* and *baba* and unintelligible voices. He could interact with friends and put rings in a column; however, he could not read, express his needs, and walk alone owing to poor balance. He had difficulties with responding to commands and being unable to form effective social relationships with others, and also with independence, movement, self-direction and social skills, money-handling skills, communication, and knowledge about numbers and time.

#### Basel

Basel was a 7-year-old boy with a diagnosis of moderate intellectual and developmental disabilities, Down’s syndrome, and speech impairment. Basel’s IQ score was 44, and his score on PLS-4 was 30 for receptive and 15 for expressive language. He could make sounds during occupational and behavior therapy. He likes several activities, such as running, vocalizing, and playing with balloons. He could insert rings in a column, build a tower of cubes, and open the door handle. As per center records, he was unable to distinguish among clothes and could not express his needs. He also had weaknesses in visual communication and imitation. He faced deficiencies in the cognitive characteristics of attention and cognition, remembering, thinking, and imagining. He was unable to form effective social relations with others and demonstrated aggression and repetitive “flutter” behavior. He had also difficulties with independence, movement, self-direction and social skills, money and language skills, bearing responsibility, and knowledge about numbers and time.

#### Swsan

Swsan was an 8-year-old girl diagnosed with moderate intellectual and developmental disabilities along with moderate cerebral palsy and speech impairment. Swsan had an IQ score of 51, and she scored 60 on PLS-4 for receptive and 20 for expressive language. She was non-verbal, but sometimes produced letters. She likes several activities, such as playing on the swing and with dolls, and music. She was able to hold the pen, color within boundaries, and draw shapes; however, she could not read and express her needs and had deficits in thinking and imagination. Nevertheless, Swsan’s cognitive characteristics of attention, focus, perception, memory, distinction, and generalization were adequately positioned. She had shortcomings in money-handling and language skills, bearing responsibility, and determining numbers and time.

#### Yousif

Yousif was a non-verbal, 7-year-old boy with a diagnosis of moderate intellectual and developmental disabilities and speech impairment. Yousif had an IQ score of 41, and his score was 60 on PLS-4 for receptive and 20 for expressive language. He likes several activities, such as jogging, yoga, sports, music, and listening to stories. He was able to classify objects, display conformity, and imitate movements. Nevertheless, he could not read and express his needs and experienced difficulty in holding a pen. He also had deficiencies in thinking and imagining. Yousif’s cognitive characteristics of attention, concentration, cognition, remembering, distinction, and generalization were well developed. He participated and interacted with others, and displayed repetitive “head-banging” behavior. He was able to perform motor skills independently, but had insufficient self-direction and social skills, money and language skills, and knowledge about numbers and time. Table 1 presents the participant characteristics.

**TABLE 1:** Participant’s characteristics.

Participant	Ryan	Basel	Swsan	Yousif
Age	6 years	7 years	8 years	7 years
Gender	Boy	Boy	Girl	Boy
Diagnosis	Intellectual and developmental delays and speech impairment	Intellectual and developmental delays with Down’s syndrome and speech impairment	Intellectual and developmental delays with moderate cerebral palsy and speech impairment	Moderate intellectual and developmental delays and speech impairment

### Setting

In Saudi Arabia, there are various patterns of providing special education and related services for students with disabilities; such as special education programs in public schools, special education institutes, and also centers that provided these services (Ministry of Education, 2016). Due to the COVID-19 pandemic, the elementary schools were closed; hence, the study was conducted in a special education center, which provided the services that the children need. The study took place in a classroom at the special education center. The participants attended the sessions conducted in this center 5 times a week from 8 a.m. to 2 p.m. They were taught in a self-contained classroom and behavioral interventions were frequently employed. There were 12 children in the classroom, who were in Grades 1 to 3. The classroom was staffed by the interventionist (the study author), one special education teacher, and two instructional aides. The study was conducted by the first author, who sat next to each participant at a teacher’s table. All sessions took place between 10 a.m. and 12 p.m. from Sundays to Thursdays. Each intervention session lasted between 8 and 14 min. The study, conducted in January–February 2021, lasted for one month and 15 days, with a total of 27 sessions being administered. The study author provided the intervention sessions and conducted data assessment for all the participants.

### Classroom Equipment

The classroom setting included several learning stations from which children could learn and acquire many skills. These stations included an independent workstation, a small group and collaboration station, an art station, a play and stories station, and a self-care station. Moreover, this classroom ensured a lack of sensory distractions in terms of an appropriate degree of paint and paintings included in the classroom. It contained a place dedicated to children’s bags, and electric shutters were covered for the safety of children. Furthermore, materials, toys, and aids of very high quality were presented to preserve the safety and health of the child; for example, clay was replaced by natural clay paste manufactured with safe tools. Each of the various main stations included in the classroom is described in more detail below.

#### Independent Workstation

This station consisted of a table and two chairs for the teacher and the child and drawers with boxes dedicated to the tools and aids used with the child during individual sessions.

#### A Small Group and Collaboration Station

This station consisted of a table shaped in the form of the letter U, and chairs for the children and teacher to ensure that the group circle is completed and the children can be taught several skills through songs, pictures, cards, and models. The use of these learning aids helps children learn to wait for their turn and facilitates participation and collaboration with peers.

#### Art Station

This station functioned to achieve calm in the classroom, develop the senses of the children and enrich their perceptions, boost enjoyment, contentment, and creativity, and build sensory and visual experiences. It was also effective in reducing some inappropriate behaviors by reducing stress and discharging negative energy. This station contained a dedicated board to display children’s drawings as an expression of their achievement.

#### Play and Stories Station

In this station, children could play freely, contributing to the strengthening of their major and minor muscles. This station also functioned to help children learn to use the surrounding tools, gain comfort, and get rid of excess negative energy. In addition, play contributes immensely to the social aspect of a child’s life, as it is through play that the child learns collaboration and communication with other children and gains self-confidence. In the story station, the child’s perceptions were expanded by presenting simple stories based on pictures and short words; moreover, given the importance of stories in the social, emotional, and psychological aspects of our lives, they work to increase the child’s linguistic vocabulary and love of exploration.

#### Self-Care Station

Owing to the importance of self-care for children for achieving independence in their life, the focus in this station was placed on training the children in the skills of bathroom use, washing hands, brushing teeth, cleaning the nose, eating, and combing hair. In general, such a classroom can also include a teacher’s table, floor toys, table toys, schedule stations, sensory table, and snack table. Accordingly, there were various tables in the classroom.

### Research Design

A multiple baseline design was implemented across all participants in this study (Horner and Baer, 1978; Kazdin, 2010; Ledford and Gast, 2018). The dependent variable was the number of correct requests using GoTalk NOW app on an iPad for each child. These requests consisted of several choices, reinforcers, and activities in the classroom setting that the child can access all items. The interventionist waits for a participant to point or gaze at an item to indicate interest, and then, the child is prompted to select the item on the iPad and is rewarded with access to the item requested. For this study, the correct responses were defined when the child, using the iPad, correctly and independently pointed and requested. As mentioned earlier, event recording was employed as a data collection method in this study. It pairs preferred items with non-preferred items so if the non-preferred item is selected, then the interventionist can certain that the selection was made by mistake or incorrect. If the child requested correctly using the iPad, the sign (√) was indicated in the data sheet, but if the child incorrectly requested using the iPad, the sign (×) was indicated in the data sheet. At the end of each session, the total number of correct responses was reordered using the preference assessment. The interventionist communicated with the parents and special education teachers, asking them to provide the ten preferred items for each participant of this study. The preferred items that were used in this study were as follows: Ryan (e.g., balls, play, small toy, and food), Basel (e.g., jumping, free play, and stuffed toys), Swsan (e.g., stories, jumping, food, and free play), and Yousif (e.g., balls, free play, jumping, and small toy).

The effects of the MLT technique paired with the GoTalk NOW app on an iPad to teach four children with intellectual and developmental disabilities to correctly request were interpreted and evaluated using a multiple baseline design across the four participants in baseline, intervention, and independent use of the GoTalk NOW app phases. The measures, such as level, trend, variability, and immediacy, were used across the three phases for all the participants. All four participants began baseline at the same time. After achieving a stable baseline path, the intervention was introduced to Ryan. After achieving a stable intervention path for Ryan, the intervention was introduced to Basel. The same procedure was followed for Swsan and Yousif.

This study was conducted with the formal approval of the local human subject committee (institutional- KSU-IRB-HE-21-404) and followed the principles of the Declaration of Helsinki.

### Materials

A variety of materials were used in this study: an iPad with an Arabic version of the GoTalk NOW app (The Attainment Company, 2020), data collection (event recording) forums to record the responses of the children.

### Procedures

#### Baseline

During baseline, the interventionist and participant sat together at the workstation with the participant’s preferred items and non-preferred items placed on the table to keep the participant motivated (Kagohara et al., 2012); however, these items were out of the reach for each participant in the classroom. The participant was given the iPad with the GoTalk NOW app displayed on the home screen with the icon “I want.” This app consisted of several preferred items’ activities, which were established from the pre-preferred items assessment for each participant. The interventionist recorded communication data for each participant using the GoTalk NOW app on iPad in a maximum of the 14-min session. If the participant requested correctly and independently, the sign (√) was given, and if the participant’s responses were incorrect, the sign (×) was given. During this session, the interventionist did not provide any prompts, reinforcements, or feedback to the participants.

#### Intervention

The intervention proceeded in phases, which included using the GoTalk NOW app and MLT technique together and then using the GoTalk NOW app independently.

##### Pairing of the GoTalk NOW App and Model, Lead, and Test Technique

During this phase, the MLT technique was paired with the GoTalk NOW app on the iPad to help participants communicate and request activities correctly and independently. The GoTalk NOW app customizes pages and activities according to the child’s preferences, interests, and abilities. When the iPad opens, the main page of the app appears, including the preferred activities of participants, customized according to the interests and preferences of each participant. The interventionist presented the iPad to the participant. If the participant requested items correctly and independently using the GoTalk NOW app on the iPad within 30 s, the access to the item was immediately given to the participant with verbal reinforcement. If the participant did not request items correctly and independently using the GoTalk NOW app within 30 s, the interventionist modeled the correct responses using several prompts such as gestures and verbal or physical prompts; the use of these prompts depended on the consequent responses of the participant. Based on the participant’s response, the interventionist initially provided gesture prompts with 10-s interval. If the participant did not request items correctly, then verbal prompts were provided. If the participant still did not request items correctly, the intrusiveness of the prompts was increased by providing physical prompts. If the participant established mastery for at least 80% of the requesting items or activities independently, the GoTalk NOW app was implemented into the next level.

##### Independent Use of the GoTalk NOW App

During this phase, only the GoTalk NOW app on the iPad was presented with other items that were from the preferred items group used in the earlier phases, and the MLT technique was removed (Marchand-Martella et al., 2004). There were no prompts (gestures and verbal or physical prompts) that modeled the correct responses for the participants. The interventionist recorded the correct and incorrect requests for each participant.

#### Interobserver Agreement

Interobserver agreement was taken in the classroom. The interventionist (the study author) and one special education teacher collected the data during the baseline and intervention sessions 50% of the time. Both the interventionist and special education teacher collected the data simultaneously for each experimental condition. The data that were collected were compared to calculate the number of agreements or disagreements between the interventionist and special education teacher. Therefore, to calculate interobserver agreement, the number of agreements was divided by the total number of agreements and disagreements, which was multiplied by 100. At the end of the study, the interobserver agreement was 98%.

#### Treatment Integrity Data

The interventionist conducted all baseline, intervention, and independent use of the GoTalk NOW app sessions and followed procedural checklists to collect treatment integrity data. The second observer was provided with the checklists and used sample videos recorded footage to check the fidelity. It was marked on the checklists the steps that performed correctly. This was assessed for 60% of all sessions. Procedural fidelity was calculated by counting the number of correctly performed steps divided by the total number of steps and multiplying by 100. The results indicated that the fidelity data for each session across all participants were 98%.

#### Social Validity

The special education teacher and instructional aides were asked to evaluate their perceptions toward the effect of “MLT” technique paired with the GoTalk NOW. All four special education teachers and instructional aides were provided social validity questionnaire to evaluate their perceptions and effectiveness of the intervention. This questionnaire included 8 questions with a 5-point Likert scale manner (5 = strongly agree, 1 = strongly disagree). For raters’ features, they hold a Bachelor’s degree in special education with about ten years of professional experience, and their age ranged from 26 to 37 years. The target behavior, acceptability, generalizability, and effects of intervention were considered. They indicated that this intervention was practical, effective, and easy-to-use method for both teachers and participants.

## Results

Data were analyzed using a combination of visual analysis and Tau-U to determine the effect size between baseline and intervention. The visual analysis provided an evaluation of level, trend, and stability (Lane and Gast, 2014) whereas a non-parametric approach for analyzing single-case data, Tau-U (Parker et al., 2011) was calculated for the dependent variable to determine the effect of the intervention. Several studies showed that the interpretation of the Tau-U is ranged as follows: weak (0.20 or less), moderate (0.21–0.59), and strong or large (0.60 or greater) (Parker et al., 2011; Vannest and Ninci, 2015; Harrison et al., 2019; Stewart and Austin, 2020). The analysis of Tau-U scores suggests that the MLT and GoTalk NOW app were strong and largely effective in developing requests, and the weighted average of Tau-U was 0.63.

### Ryan

**FIGURE 1:**
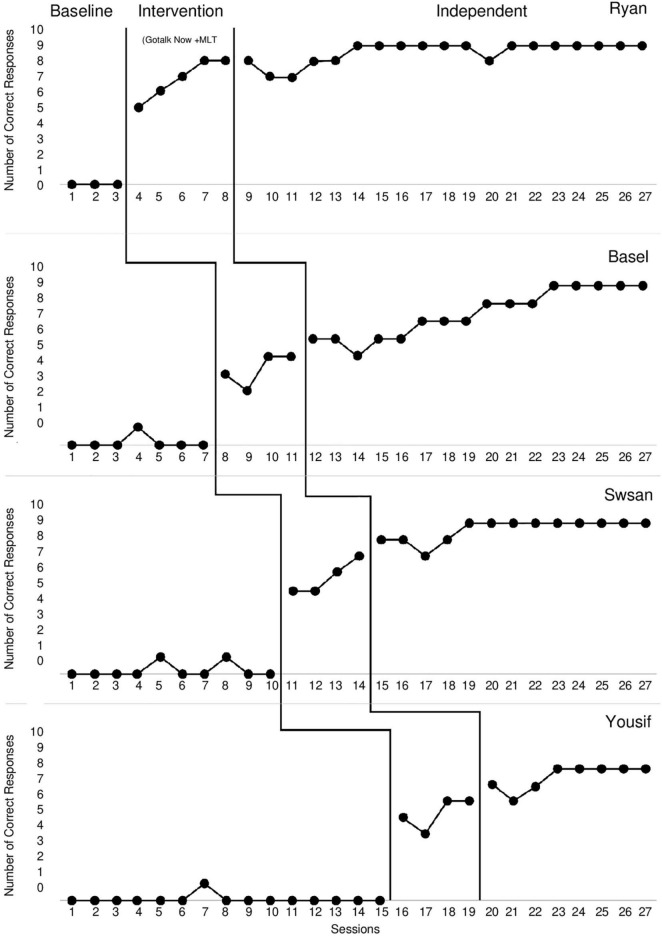
The number of correct requests by each participant on the iPad for the baseline, intervention (MLT technique paired with the GoTalk NOW app), and (independent use of the GoTalk NOW app) phases.

The results for Ryan across the baseline, intervention, and independent use of the GoTalk NOW app phases are shown in the top panel of Figure 1. Ryan did not request correctly during the baseline phase. Hence, there was no level, trend, or variability during the baseline phase. During the baseline, the mean score of possible correct requests was 0. At the beginning of the intervention phase, the data showed a visually positive change in level compared with the baseline session. There was a positive trend, low variability, and positive immediate and pronounced change. The mean score of possible correct requests in the intervention phase, which involved pairing the MLT technique with the GoTalk NOW app, was 6.8) range: 5–8). Ryan did request correctly and independently during the independent use of the GoTalk NOW app phase. The mean score of possible correct requests in the independent use of the GoTalk NOW app phase, which involved the independent use of the GoTalk NOW app, was 8.57) range: 7–9), with no prompts provided to Ryan. Ryan reached a plateau after eight sessions.

### Basel

The results for Basel across the baseline, intervention, and independent use of the GoTalk NOW app phases are shown in the second panel of Figure 1. Basel slightly and spontaneously requested correctly during the baseline phase. During the baseline, the mean score of possible correct requests was 0.14) range: 0–1), with less variability. At the beginning of the intervention phase, the data showed a visually positive change in level compared with the baseline session. There was a positive trend, less variability, and positive immediate and pronounced change. The mean score of possible correct requests in the intervention phase was 4.25) range: 3–5). Basel did request correctly and independently during the independent use of the GoTalk NOW app phase. The mean score of possible correct requests in the independent use of the GoTalk NOW app phase was 7.43 (range: 5–9), with no prompts provided to Basel. Basel reached a plateau after 11 sessions.

### Swsan

The results for Swsan across the baseline, intervention, and independent use of the GoTalk NOW app phases are shown in the third panel of Figure 1. Swsan slightly and impulsively requested correctly during the baseline phase. During the baseline, the mean score of possible correct requests was 0.2 (range: 0–1), with low variability. At the beginning of the intervention phase, the data showed a visually positive change in level compared with the baseline session. The mean score of possible correct requests in the intervention phase was 5.75) range: 5–7). Swsan did request correctly and independently during the independent use of the GoTalk NOW app phase. The mean score of possible correct requests in the independent use of the GoTalk NOW app phase was 8.61) range: 7–9), with no prompts provided to Swsan. Swsan reached a plateau after 14 sessions.

### Yousif

The results for Yousif across the baseline, intervention, and independent use of the GoTalk NOW app phases are shown in the bottom panel of Figure 1. Yousif spontaneously requested correctly during the baseline phase. During the baseline, the mean score of possible correct requests was 0.06) range: 0–1), with less variability. At the beginning of the intervention phase, the data showed a visually positive change in level compared with the baseline phase. There was a positive trend, less variability, and positive immediate and pronounced change. The mean score of possible correct requests in the intervention phase was 5.25) range: 4–6). Yousif did request correctly and independently during the independent use of the GoTalk NOW app phase. The mean score of possible correct requests in the independent use of the GoTalk NOW app phase was 7.5) range: 6–8), with no prompts provided to Yousif. Yousif reached a plateau after 19 sessions.

## Discussion

This study investigated the effectiveness of the MLT error correction technique paired with the GoTalk NOW app on an iPad to teach four children with intellectual and developmental disabilities to correctly request. The findings of this study indicated that all four participants made a clear progress using the iPad, and implementing the MLT technique was successful in helping the participants request correctly and independently. These results are consistent with those of previous studies (Peterson et al., 2008; Achmadi et al., 2012; Dundon et al., 2013; Ward et al., 2013; Hill and Flores, 2014; Clinical Innovation and Governance, 2016; Alzrayer et al., 2020), which have indicated that children with intellectual and developmental disabilities are able to request for activities using the GoTalk NOW app on the iPad.

In all four participants, the MLT error correction technique paired with the GoTalk NOW iPad app was effective at teaching children with intellectual and developmental disabilities. The findings replicated the results of other studies regarding the MLT error correction technique paired with the GoTalk NOW app. Dundon et al. (2013) found positive outcomes and an increase in correct and independent requesting when the MLT technique and GoTalk NOW app were used together. In addition, Ward et al. (2013) indicated that the children’s use and knowledge of making independent requests increased when using the GoTalk NOW app as a commutation strategy on the iPad.

Based on the results of the study participants, a positive effect on their knowledge of and skills relative to correctly making independent requests was found after they used the iPad communication app. This suggests that the implementation by teachers of iPad apps in the process of communicating with children who have intellectual and developmental disabilities and who have complex communication difficulties leads to them making independent requests and communicating correctly with others. This is confirmed by previous studies on the effectiveness of using these applications as augmentative or alternative communication options with similar children (Dundon et al., 2013; Lorah et al., 2013; Ward et al., 2013; and Hill and Flores, 2014).

The findings of this study also suggested that the GoTalk NOW app generalized across the classroom teacher for the four participants. These participants maintained their use of the GoTalk NOW app in independent use of the GoTalk NOW app sessions, suggesting that the removal of the MLT technique did not affect their independent and correct requests, which was reported in some studies (Dundon et al., 2013; Ward et al., 2013). Overall, the findings from this study can be seen as helping to extend the literature by indicating that adding additional techniques (e.g., the MLT technique) to GoTalk NOW app interventions could enhance the functional communication skills of children with intellectual and developmental disabilities.

### Implications for Practice

The procedures that were implemented in this study could be practical and effective for children with intellectual and developmental disabilities; they also could be seen as an easy way and clear to use by teachers in classrooms (McLaughlin et al., 2011; Boesch et al., 2013; Dundon et al., 2013; Lorah et al., 2013). Therefore, a functional relationship could be demonstrated between the GoTalk NOW app on the iPad and requests made correctly and independently by the participants. For example, immediacy was found for all participants using the GoTalk NOW app across the sessions.

The study also extends the implementation of the MLT error correction technique paired with the GoTalk NOW app on an iPad. Although this association has been shown in several studies (Peterson et al., 2008; Herberg et al., 2011; Dundon et al., 2013; Mangundayao et al., 2013), this study focused on tablets or digital devices (GoTalk NOW app) on the iPad rather than on the picture exchange communication system or direct instruction flashcards (Lorah et al., 2013; Hill and Flores, 2014; Alzrayer et al., 2020). This study focused on using apps on the iPad for children with intellectual and developmental disabilities even though most studies have focused on participants with autism spectrum disorders rather than on those with intellectual and developmental disability or intellectual and developmental disabilities (Dundon et al., 2013; Ward et al., 2013; Genc-Tosun and Kurt, 2017; Vlachou and Drigas, 2017).

### Limitations and Future Directions

This study had a few limitations. First, it was carried out during the time of the coronavirus disease pandemic of 2019, which negatively impacted the interaction between the interventionist and participants. The sessions could have been more effectively implemented based on national health procedures, which were suspended owing to the pandemic. Second, the study had a small sample of four children with intellectual and developmental disabilities, affecting the generalizability of the results. Hence, there is a need for future studies, such as experimental and quasi-experimental research designs using a larger sample size to measure the effects of the GoTalk NOW app on the iPad with control and experimental groups. Third, some participants displayed inappropriate behaviors associated with their disabilities; for instance, disruptive behavior displayed by Swsan negatively influenced the implementation and use of the iPad, which affected the interventionist while teaching her. Therefore, the interventionist needed more time to use the iPad in a suitable and appropriate way. Fourth, this study did not follow up with the children a few months after the intervention, which would have revealed whether the new skills learned by the children had been retained. Hence, there is a need for future studies to measure abilities for a longer time period following the intervention (i.e., measure long-term effects). Finally, despite the effectiveness of using the GoTalk NOW app for requesting independently and correctly, its use was limited at the participants’ homes, which might have led to some loss of skill learning; this requires that families too, according to their capabilities, use the iPad to communicate with their children. In general, future studies are needed to determine the effectiveness of using the GoTalk NOW app and the MLT technique for requesting independently and correctly in new settings, such as general education classroom settings and home. Future studies also needed to include participants of various ages and educational stages. Future studies should be conducted to know the teachers’ and parents’ perspectives toward using the GoTalk NOW app and the MLT technique intervention for requesting independently and correctly.

## Conclusion

This study could provide a practical, effective, and easy-to-use method that might be used by special education teachers to teach children with intellectual and developmental disabilities. It contributes to the body of empirical research on the MLT technique paired with the GoTalk NOW app on an iPad. Additionally, this study is the first in the Arab world to experimentally evaluate the effects of the MLT technique paired with the GoTalk NOW app (Arabic version) on an iPad to teach four children with intellectual and developmental disabilities to correctly request. Future research will be needed to compare the effectiveness of the GoTalk NOW app with other apps in teaching children with intellectual and developmental disabilities to request correctly and independently and helping them acquire functional and academic skills. Future research should also examine the extent to which the GoTalk NOW app (Arabic version) on an iPad can be generalized across various other children, settings, and items.

## Data Availability Statement

The raw data supporting the conclusions of this article will be made available by the authors, without undue reservation.

## Ethics Statement

This study was conducted with the formal approval of the local human subject committee (institutional- KSU-IRB-HE-21-404). Written informed consent to participate in this study was provided by the participants’ legal guardian/next of kin. Written informed consent was obtained from the individual(s) for the publication of any potentially identifiable images or data included in this article.

## Author Contributions

NA contributed to conception and design of the study, organized the database and performed the statistical analysis, and wrote the first draft of the manuscript and wrote the final sections of the manuscript.

## Conflict of Interest

The author declares that the research was conducted in the absence of any commercial or financial relationships that could be construed as a potential conflict of interest.

## Publisher’s Note

All claims expressed in this article are solely those of the authors and do not necessarily represent those of their affiliated organizations, or those of the publisher, the editors and the reviewers. Any product that may be evaluated in this article, or claim that may be made by its manufacturer, is not guaranteed or endorsed by the publisher.
