# Supplementary material for: Using the Model, Lead, and Test Technique and “GoTalk NOW” App to Teach Children With Intellectual and Developmental Delays to Correctly Request
Source: Front Psychol. 2022 Feb 9;12:811510. doi: 10.3389/fpsyg.2021.811510 (PMC8865384; doi:10.3389/fpsyg.2021.811510)

Supplementary Material

Independent Workstation

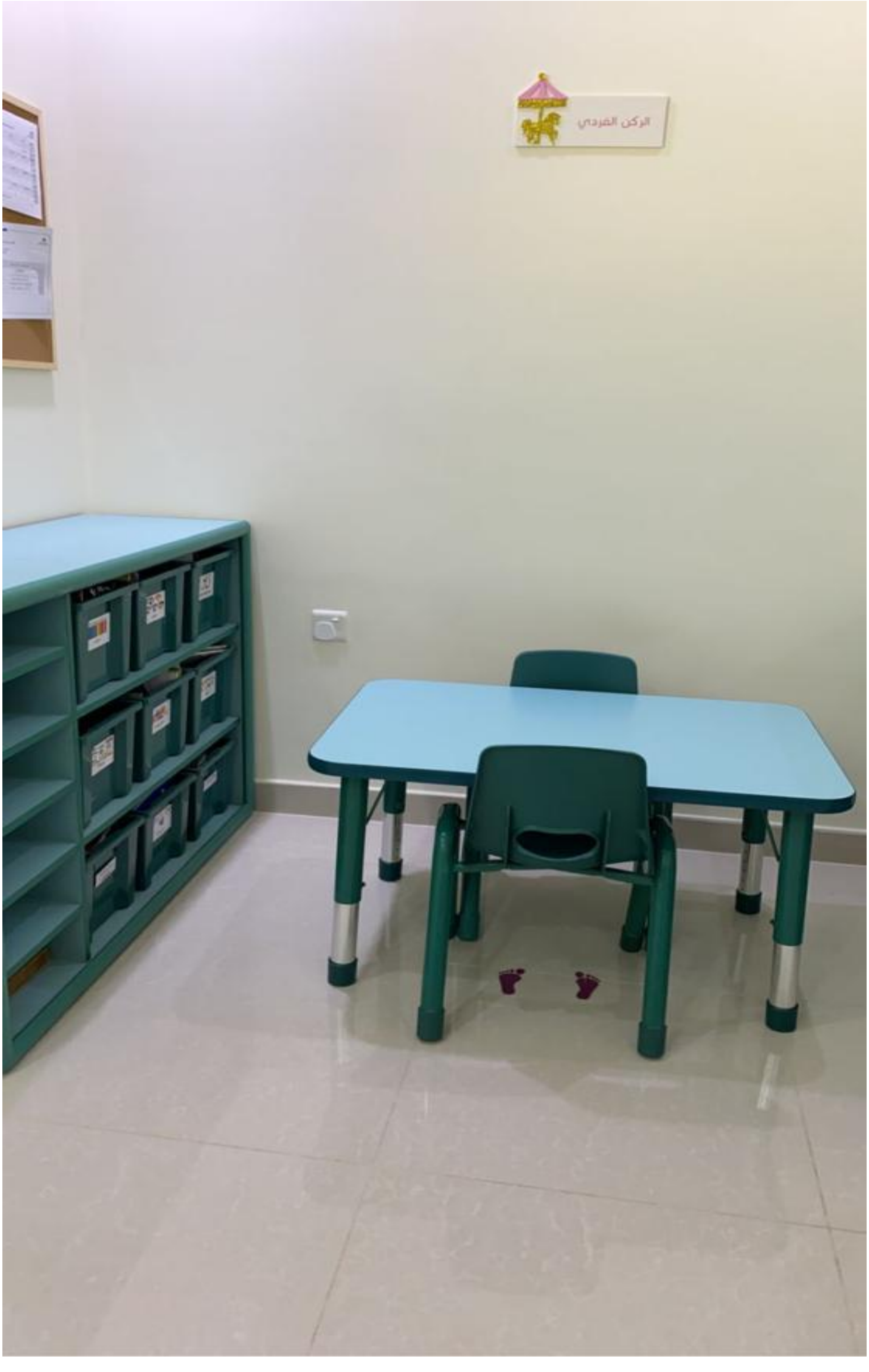

Supplementary Material

A Small Group and Collaboration Station

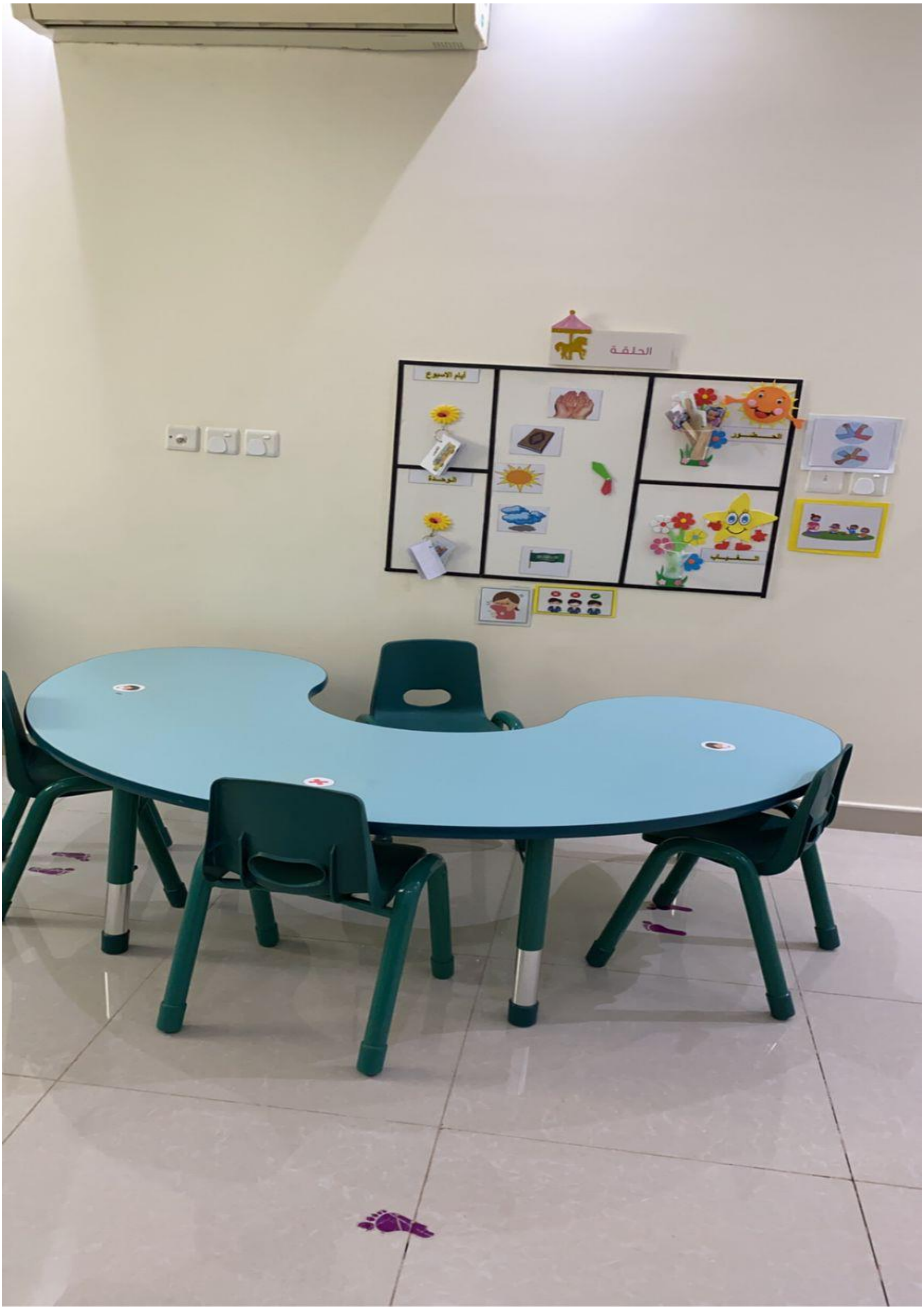

Supplementary Material

Art Station

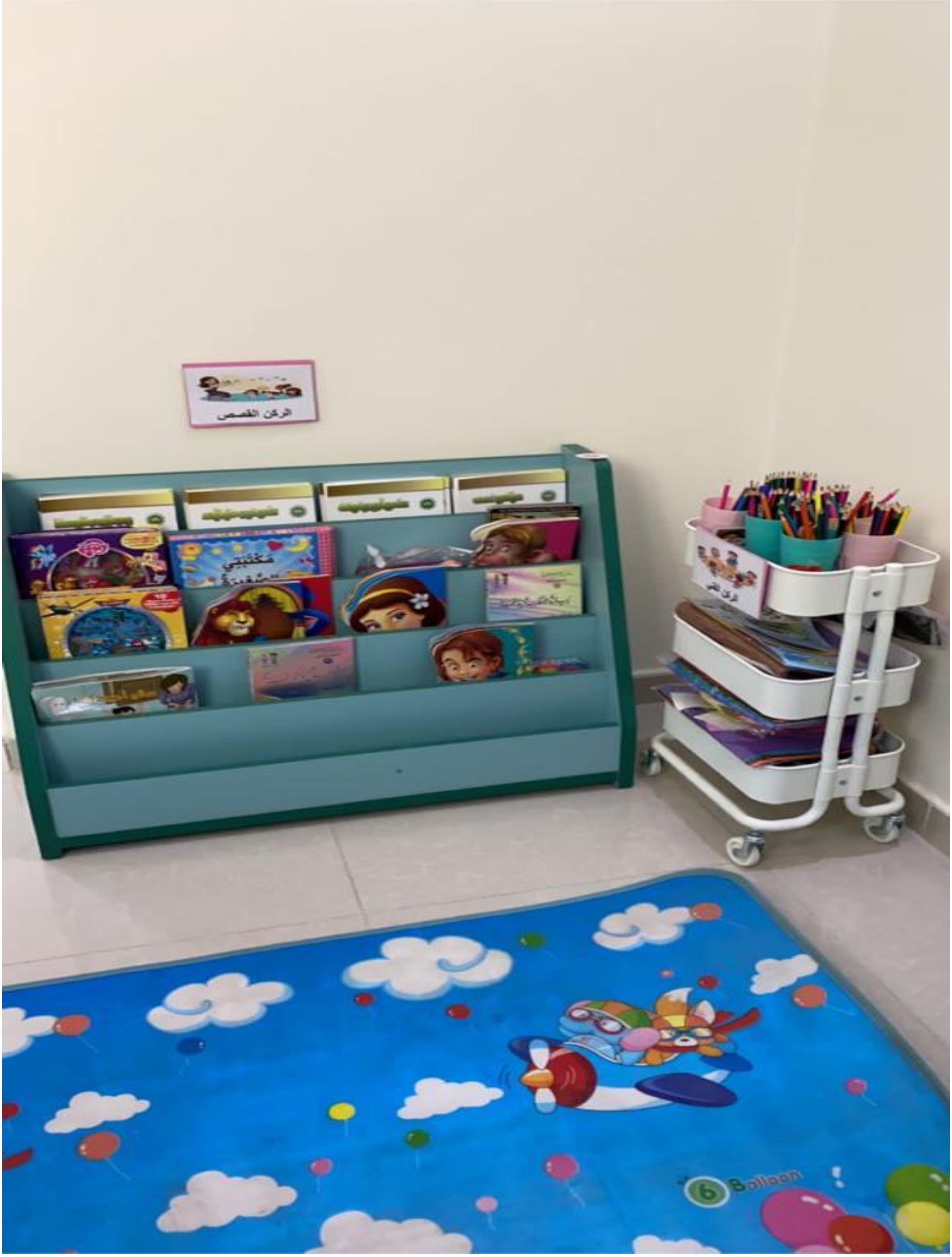

Supplementary Material

Play and Stories Station

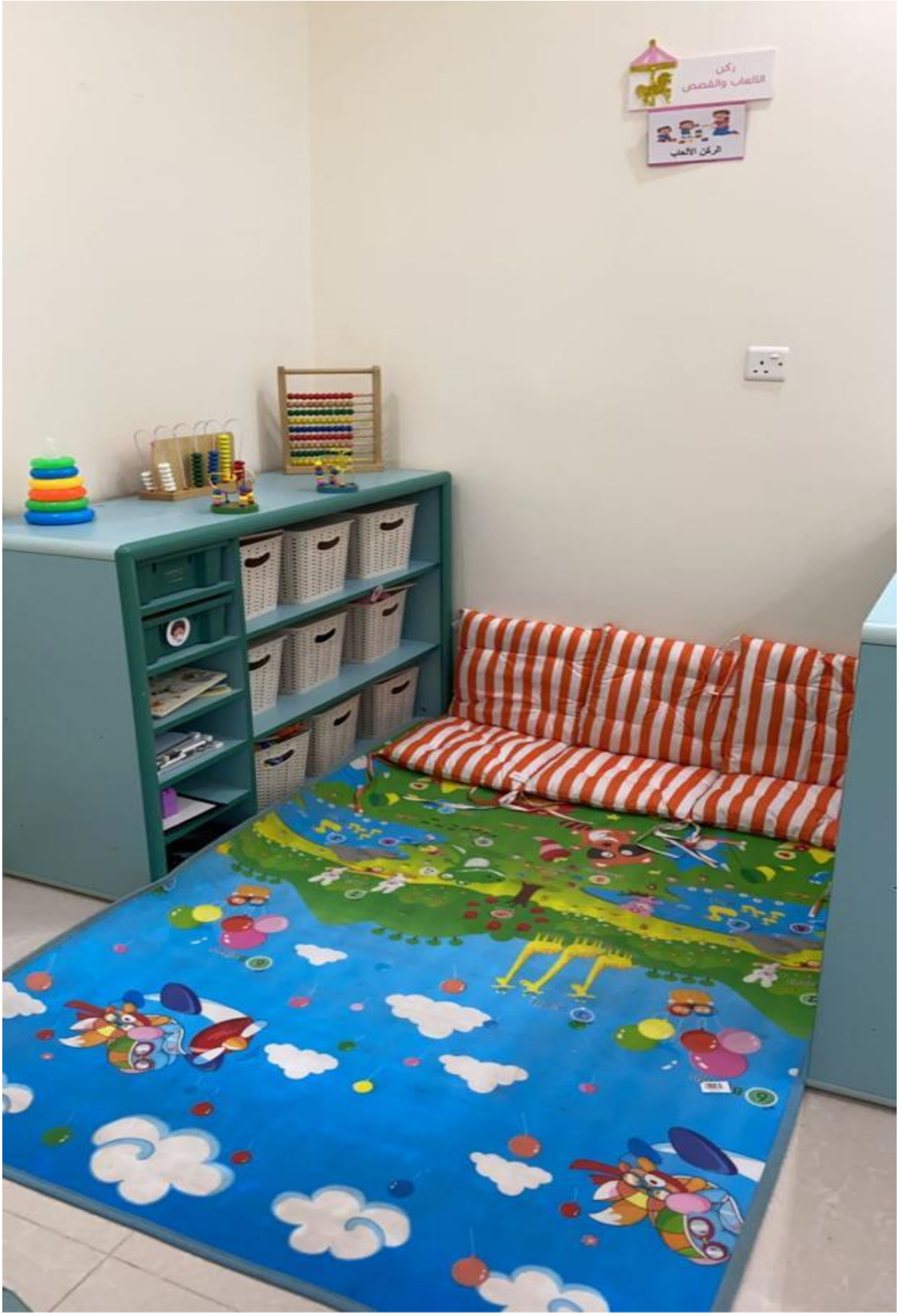

Supplementary Material

Self-care Station

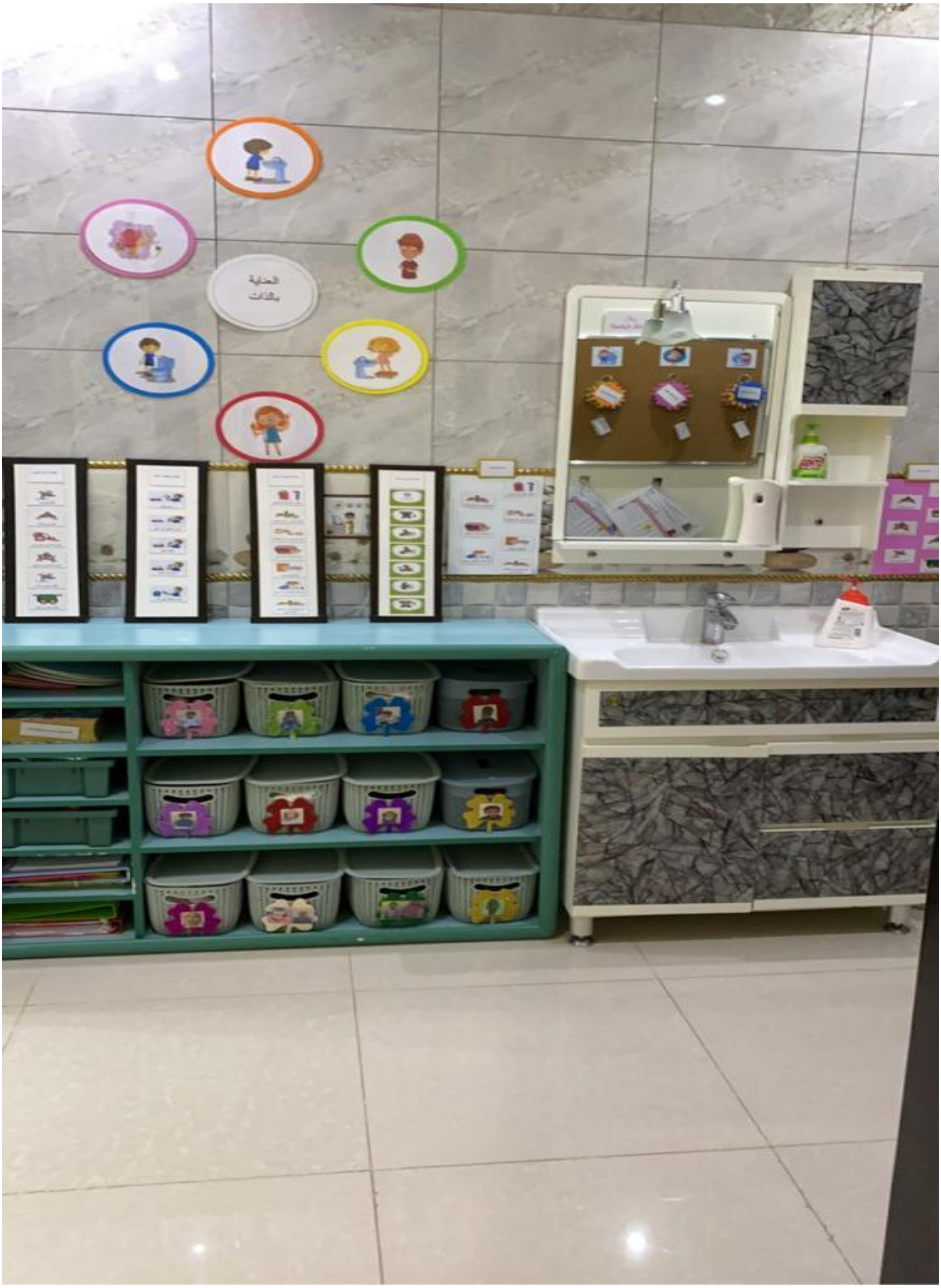

Supplement: Supplementary file 1 [file Data_Sheet_1.PDF]
