# Supplementary material for: Using the Model, Lead, and Test Technique and “GoTalk NOW” App to Teach Children With Intellectual and Developmental Delays to Correctly Request
Source: Front Psychol. 2022 Feb 9;12:811510. doi: 10.3389/fpsyg.2021.811510 (PMC8865384; doi:10.3389/fpsyg.2021.811510)

GoTalk Now App Pictures

The original version of GoTalk® NOW

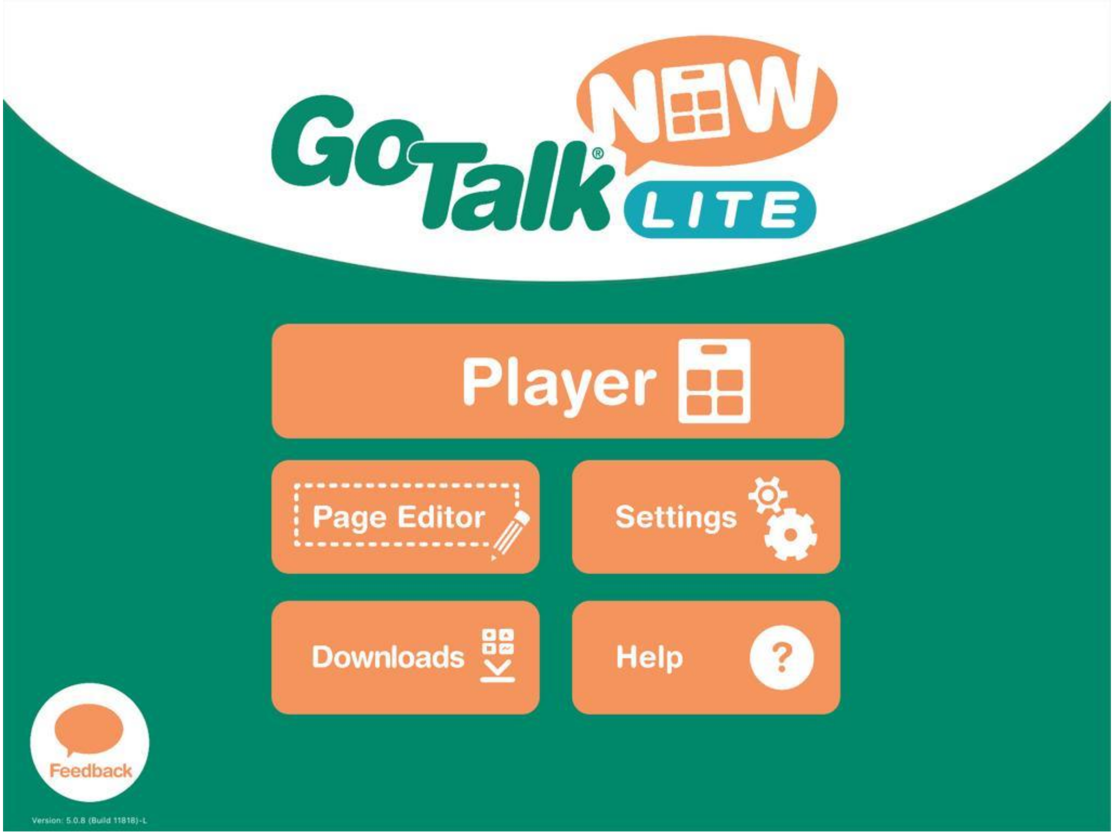

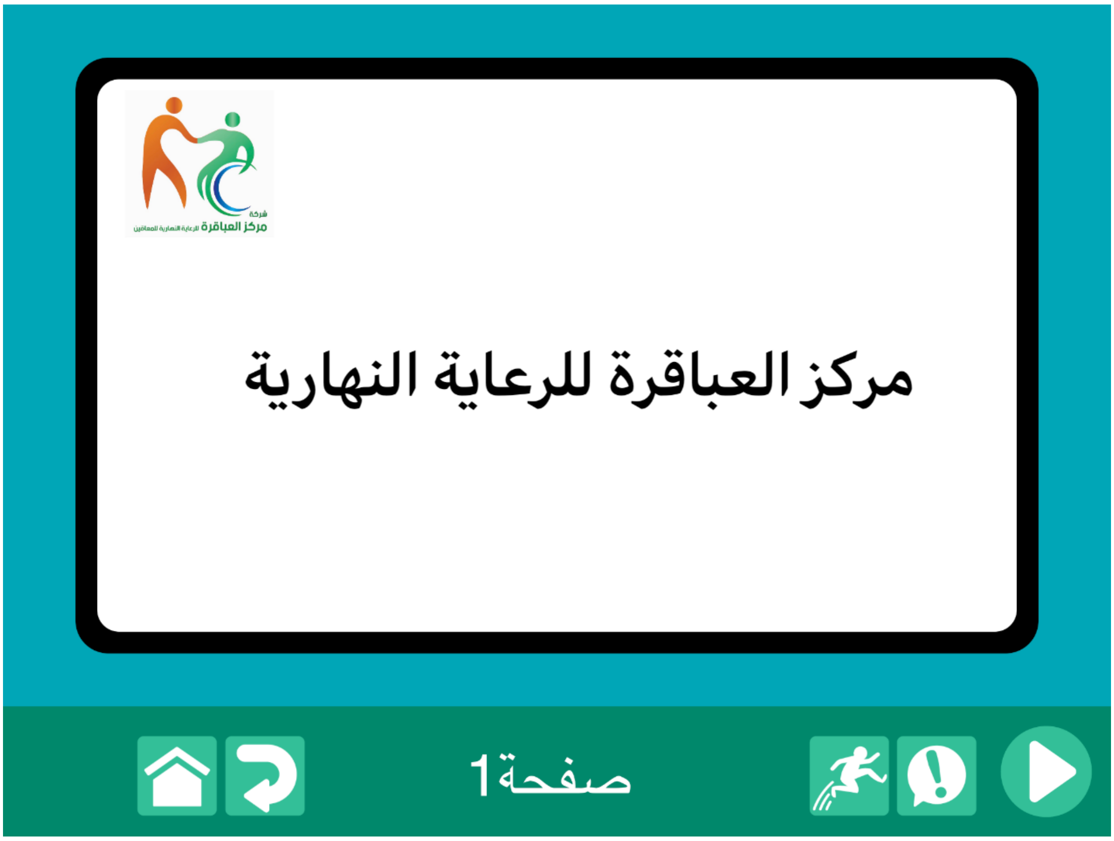

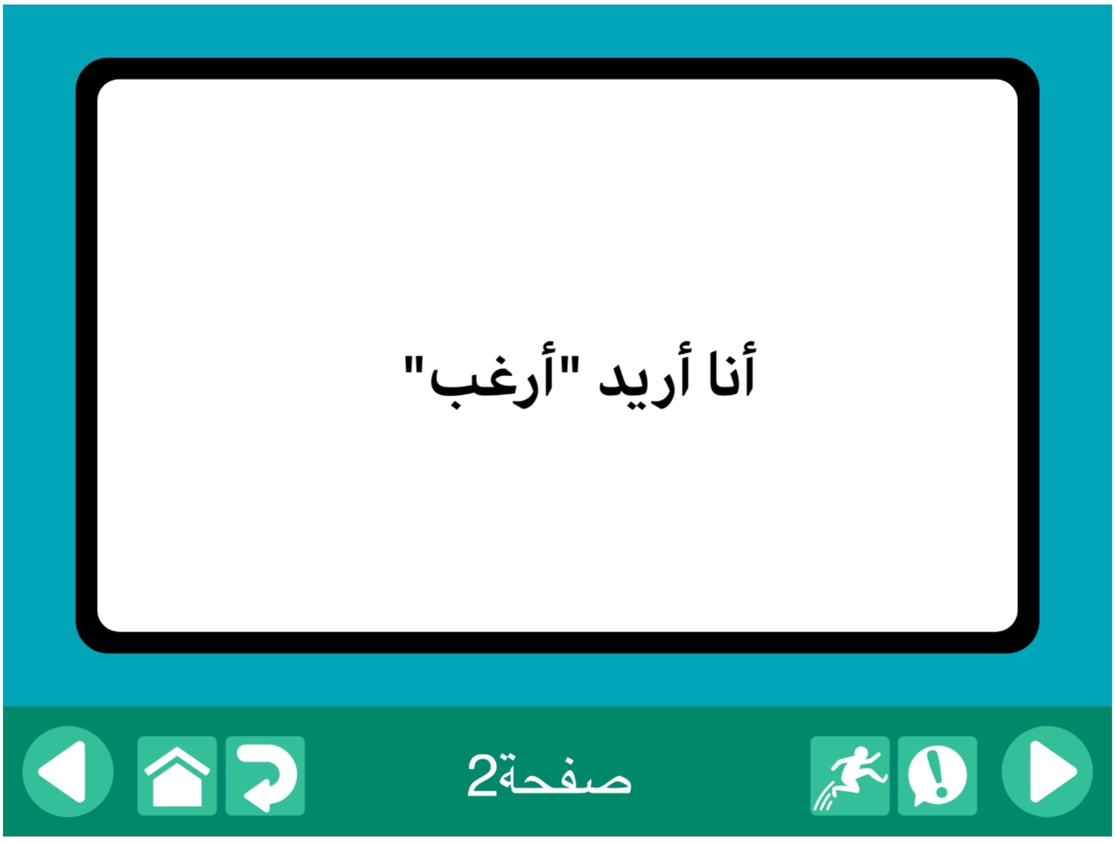
أنا أريد "أرغب"
صفحة2

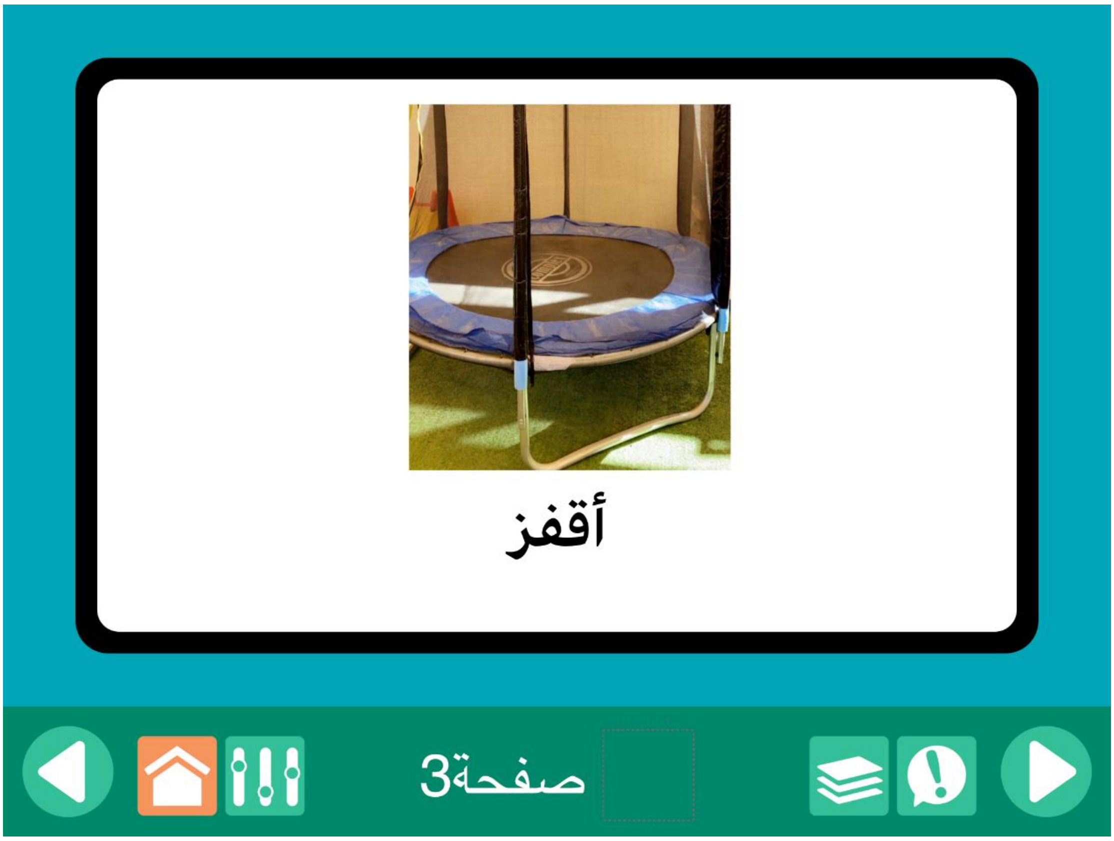
أقفز
صفحة3

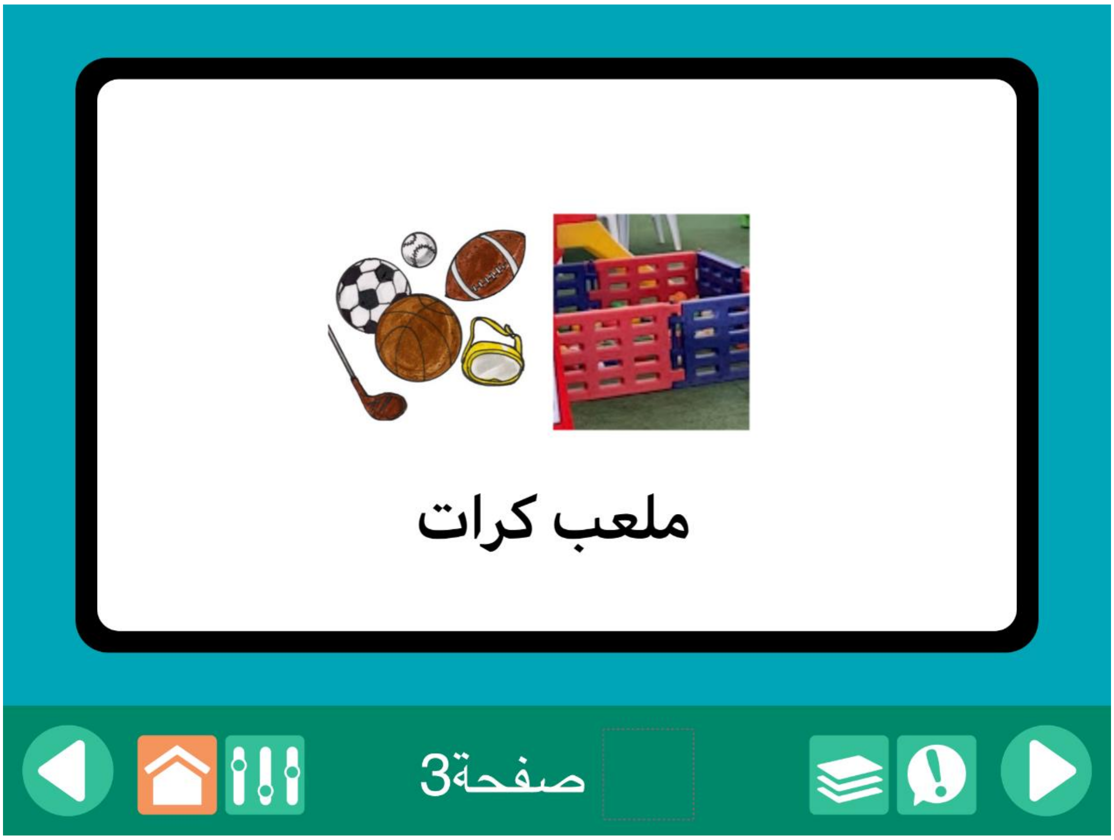
ملعب كرات
صفحة3

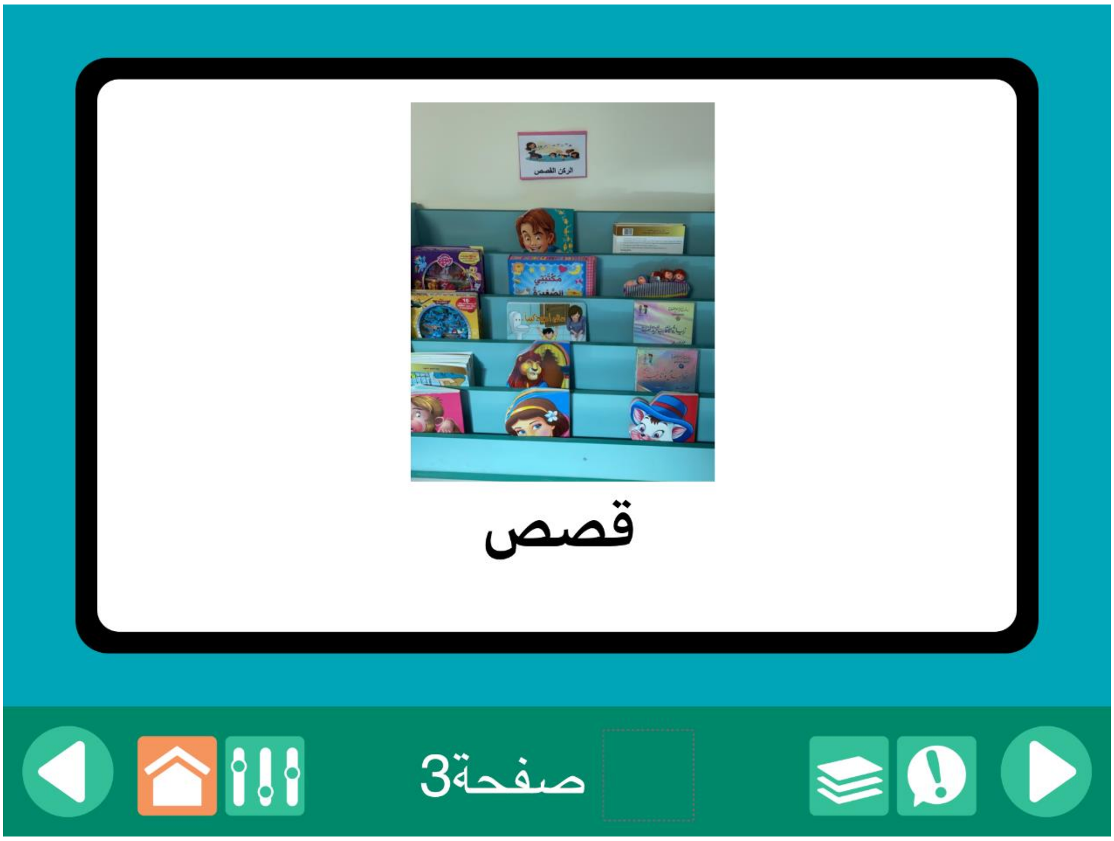
قصص
صفحة3

GoTalk Now App Pictures

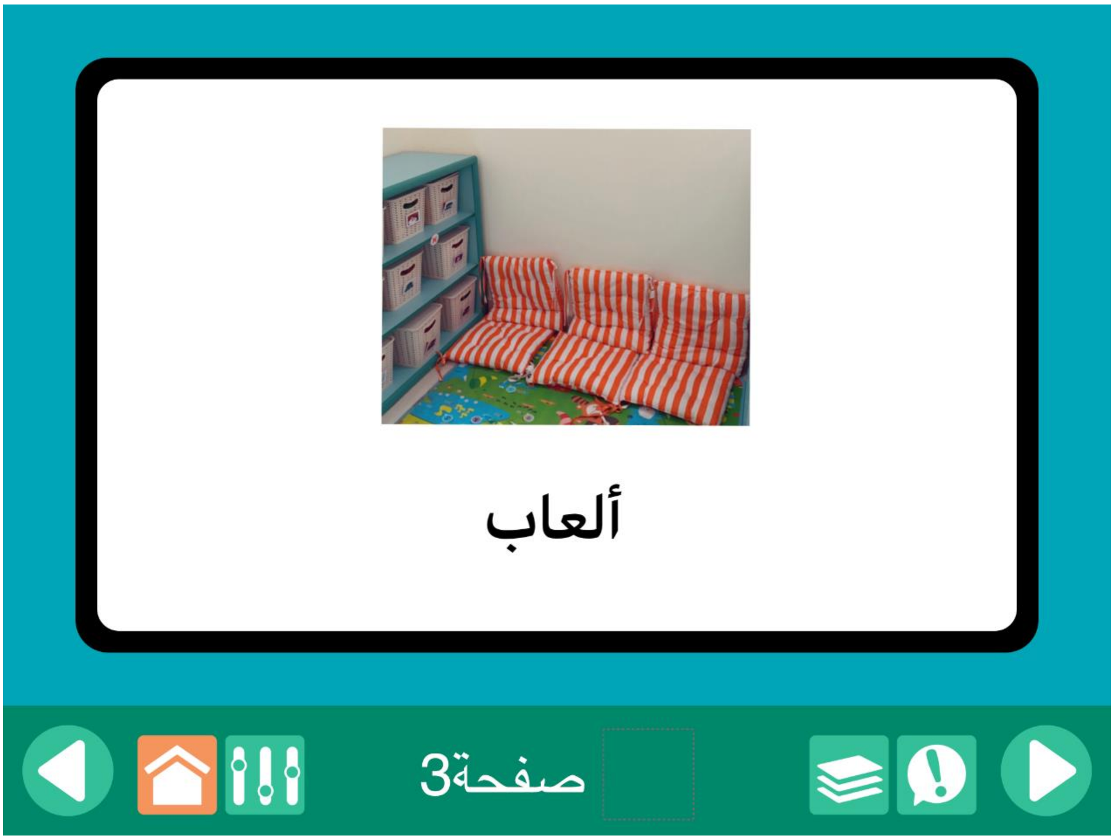

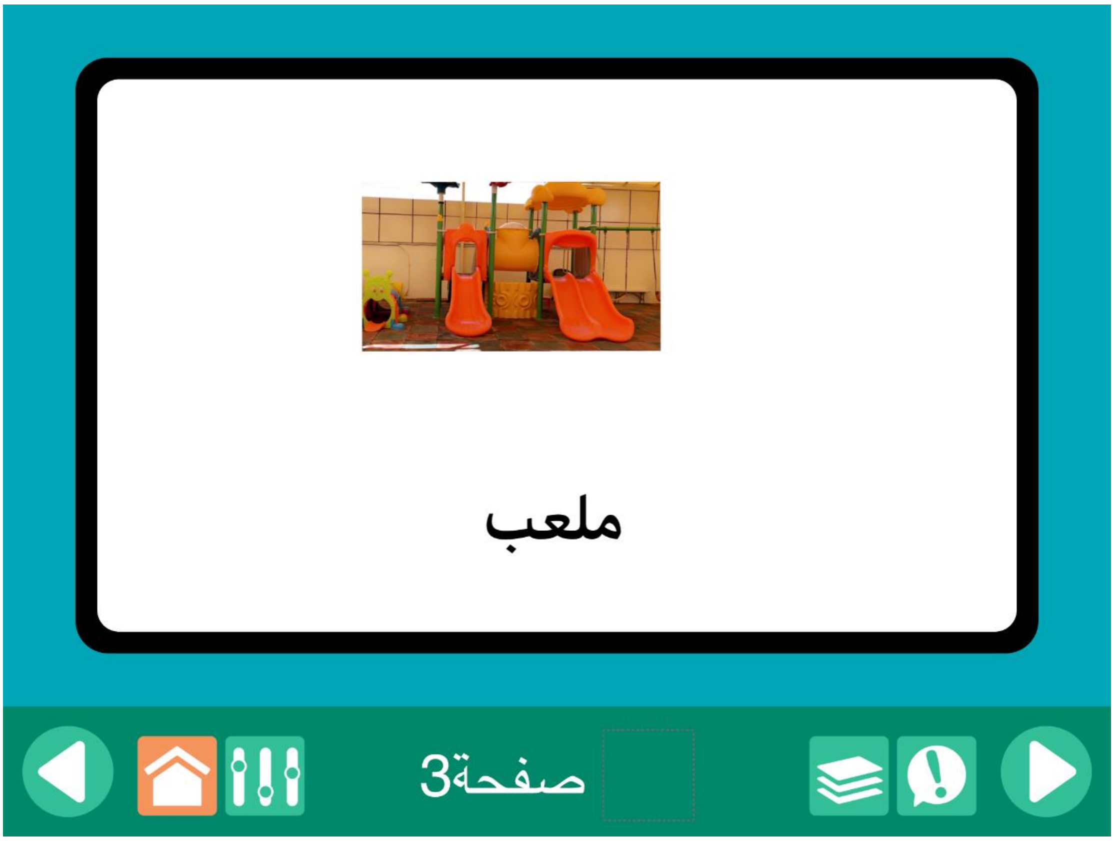

Supplement: Supplementary file 2 [file Data_Sheet_2.PDF]
